# A Forward-Genetic Screen and Dynamic Analysis of Lambda Phage Host-Dependencies Reveals an Extensive Interaction Network and a New Anti-Viral Strategy

**DOI:** 10.1371/journal.pgen.1001017

**Published:** 2010-07-08

**Authors:** Nathaniel D. Maynard, Elsa W. Birch, Jayodita C. Sanghvi, Lu Chen, Miriam V. Gutschow, Markus W. Covert

**Affiliations:** 1 Department of Bioengineering, Stanford University, Palo Alto, California, United States of America; 2 Department of Chemical Engineering, Stanford University, Palo Alto, California, United States of America; Université Paris Descartes, INSERM U571, France

## Abstract

Latently infecting viruses are an important class of virus that plays a key role in viral evolution and human health. Here we report a genome-scale forward-genetics screen for host-dependencies of the latently-infecting bacteriophage lambda. This screen identified 57 *Escherichia coli* (*E. coli*) genes—over half of which have not been previously associated with infection—that when knocked out inhibited lambda phage's ability to replicate. Our results demonstrate a highly integrated network between lambda and its host, in striking contrast to the results from a similar screen using the lytic-only infecting T7 virus. We then measured the growth of *E. coli* under normal and infected conditions, using wild-type and knockout strains deficient in one of the identified host genes, and found that genes from the same pathway often exhibited similar growth dynamics. This observation, combined with further computational and experimental analysis, led us to identify a previously unannotated gene, *yneJ*, as a novel regulator of *lamB* gene expression. A surprising result of this work was the identification of two highly conserved pathways involved in tRNA thiolation—one pathway is required for efficient lambda replication, while the other has anti-viral properties inhibiting lambda replication. Based on our data, it appears that 2-thiouridine modification of tRNA^Glu^, tRNA^Gln^, and tRNA^Lys^ is particularly important for the efficient production of infectious lambda phage particles.

## Author Summary

In this study, we took advantage of a new genetic resource for *E. coli* mutants to screen for previously undiscovered lambda phage host-dependencies. We then assessed the dynamics of infection in these different *E. coli* mutants and applied a mathematical model of infection in an attempt to further classify the role of these novel interactions. This model-driven approach to biological discovery led us to identify the previously uncharacterized gene *yneJ* as a regulator of *lamB* gene expression. In addition, we identified two highly conserved pathways involved in post-transcriptional modification of tRNA—one pathway was required for efficient lambda replication, while the other has anti-viral properties inhibiting lambda replication. This finding is important as it illustrates a new potential anti-viral strategy that could be applied broadly to other viruses.

## Introduction

Viral infections present a deadly paradox: in spite of the apparent simplicity of the viral genome, the complexity of the infection process has, for the most part, thwarted our attempts to prevent or cure it. Viral infections pose a serious threat to populations in both developing and developed countries. Additionally, viral infection is a serious problem for the bioprocessing industry, threatening production of items ranging from food to pharmaceuticals [1]. Increasing our understanding of viral infection would therefore have a major impact on human health, industry, and quality of life.

One resolution of this paradox is that the complexity of infection is not limited by the scope of the viral genome, but by the host machinery that the virus must commandeer in order to replicate. Recently, several genome-scale experimental studies have sought to identify these host-dependencies in viral replication. Research groups studying HIV [2]–[4], Influenza virus [5], [6], West Nile virus [7], Hepatitis C [8], [9], yeast virus [10], and T7 bacteriophage [11] have made use of newly constructed host knockout or siRNA knockdown libraries, in order to perturb the host and identify host dependencies. These forward-genetic screens have identified hundreds of host factors involved in viral infection and have provided a greater appreciation for the host's contribution to viral infection.

Today, the best-characterized model of viral infection remains bacteriophage lambda and its host—*E. coli*. Lambda is a temperate phage with two possible outcomes upon cell entry. In lytic growth (also known as productive growth), phage quickly replicate and lyse the cell, releasing new phage particles into the surrounding environment. In lysogenic growth, the injected phage DNA integrates into the *attB* site of *E. coli* genome and becomes a prophage [12]. The inserted prophage lies dormant until a later time, when upon induction the prophage genome excises itself from the host genome and begins productive growth. The determinants of lytic versus lysogenic growth appear to depend on several factors, such as multiplicity of infection [13], temperature [14], [15], and host cell physiology (e.g., nutrient state and size) [16], [17]. The lambda-*E. coli* system has also been a central player in elucidating and helping to understand host-virus interactions. Many genetic screens have been used to understand the infection phenotypes of different virus and host mutants [18]. These studies have greatly increased our knowledge pertaining to viral infection.

In this study, we focused on determining the interactions between *E. coli* and lambda phage during the infection process. We began with a forward-genetic screen to identify the *E. coli* genes whose absence results in a significantly reduced infection by phage lambda. We then performed higher resolution measurements of the infection time course for each gene and used a combination of bioinformatics and mathematical modeling in an effort to more rapidly identify likely roles in the lambda lifecycle.

## Results

### Lambda infectivity screen

**Figure 1:**
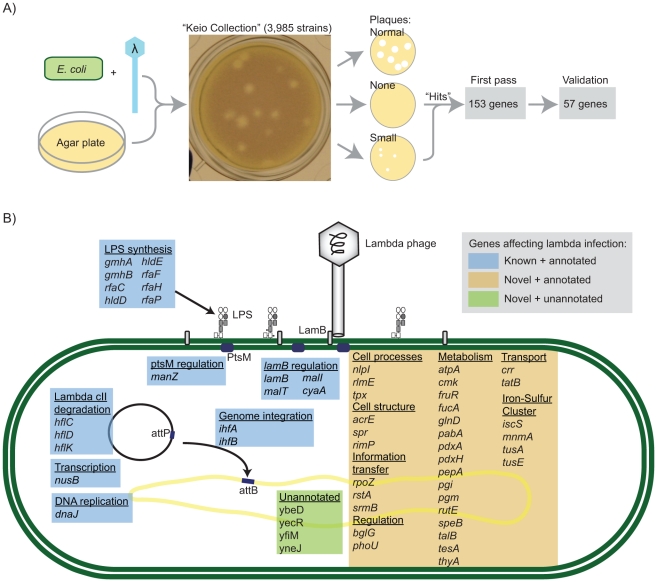
A large-scale screen to determine the *E. coli* genes required for phage lambda infection. (A) Schematic of the screen procedure. All of the strains in the “Keio Collection” were infected with phage lambda. Strains that exhibited significantly fewer or smaller plaques than the K-12 WT control were considered “hits”. (B) The results of the screen, grouped by functional category. The colored boxes indicate the annotation status of the gene and whether it had been previously associated with lambda phage infection (see legend).

Our screen to determine *E. coli* genes involved in lambda phage infection made use of the “Keio Collection”, an in-frame single-gene knockout strain collection, which contains 3,985 strains corresponding to all the genes which are non-essential during growth in rich medium [19] (see Figure 1A). We grew each knockout strain, as well as the “wild-type” K-12 MG1655 strain (K-12 WT), together with lambda phage on an agar plate with nutrient broth (NB) and 24 hours later assessed the resulting plaque morphology. In the first pass, 152 knockout strains appeared to affect lambda replication efficiency, producing either no visible plaques or smaller plaques relative to K-12 WT. All of the strains that appeared to inhibit phage replication, along with an additional 88 strains that were difficult to assess—primarily due to lawn defects—were considered further in two replicate experiments.

In all, 57 strains were identified with significantly different plaque morphology from K-12 WT (see Figure 1B and Table S1). The genes corresponding to these strains fall into three categories with respect to annotation: (i) genes with a known role in lambda infection, (ii) well-characterized genes whose products had nevertheless not been implicated in lambda infection, and (iii) unannotated genes.

**Figure 2:**
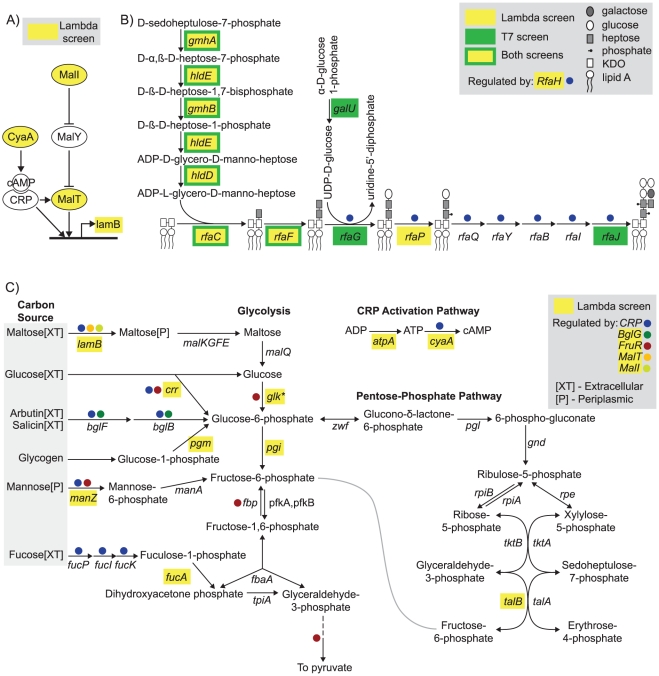
Schematics of *E. coli* pathways and networks involved in lambda infection. The genes found in this screen are highlighted in yellow. (A) The *lamB* gene and several genes governing its transcriptional regulation. (B) Biosynthesis of the LPS inner core. Several genes shown here were also identified in a screen for T7 phage infectivity (highlighted in green). (C) Several entry points to central carbon metabolism, with corresponding transcriptional regulation. The asterisk indicates that *glk* was not among the original 57 “hits” found in the screen but is highlighted here because only one small plaque was found in the assay.

The first group—well-known *E. coli* genes involved in lambda infection—included 19 genes. These include genes involved with lambda transport including *lamB*, which encodes a membrane protein required for the phage to bind *E. coli* [20], as well as transcriptional regulators of *lamB: malT* [21], *malI* [22] and *cyaA* [23], [24] (see Figure 2A). The inner membrane transporter *manZ* is part of a mannose PTS permease that is thought to be used by lambda phage to transport its genome into the cytoplasm [25], [26]. *cyaA* plays a dual role in lambda infection as it regulates expression of *lamB* and is involved in the lysis-lysogeny decision [24], [27].

In addition to *cyaA*, other genes involved in the lysis-lysogeny switch were found, including the proteases encoded by *hflC*, *hflK* and *hflD*. The FtsH-HflKC complex contributes to *E. coli* lysis by degrading the cII lambda transcription factor [28]. HflD directly interacts with cII, facilitating its degradation as well as disrupting its DNA binding ability [29], [30]. *ihfA* and *ihfB* were also found in the screen, and their gene products form a complex called integration host factor (IHF), which has been shown to induce sharp bends in DNA and is required for the integration of the prophage into the *E. coli* genome [31]. IHF has also been shown to play a role in phage DNA maturation [32].

The chaperone DnaJ contributes to a complex that works to destabilize the lambda P-DnaB complex bound to the *ori* site, thus allowing the DNA to be unwound and replicated [33]. The antiterminator gene *nusB* is known to play an important role in transcriptional dynamics of phage infection [34].

*gmhA*, *gmhB*, *hldD*, *hldE*, *rfaC*, *rfaF*, *rfaH*, and *rfaP* are all involved in the synthesis of the inner core of lippopolysaccharide (LPS) and were shown to affect the ability of phage to infect each strain (see Figure 2B). ADP-L-glycero-ß-D-manno-heptose is a key precursor to the inner core of LPS and is synthesized from D-sedoheptulose-7-phosphate by the gene products of *gmhA*, *gmhB*, *hldD*, and *hldE*. The heptose is then covalently bound to the KDO-Lipid A region by *rfaC*. All of the genes in the pathway, up to and including *rfaP* (with the exception of *rfaG*, whose activity may be replaced by *rfaJ*), showed reduced infectivity in our screen. This corresponds to the point in the pathway where the first heptose is phosphorylated. These eight LPS biosynthesis genes represent ∼73% of the genes found in a recent screen for *E. coli* genes required for T7 infection [11].

Previous work has shown that K-12 strains with varied LPS compositions have altered lambda receptor activity [35], [36]. Two possible explanations of this observation have been suggested. One study showed that mutations for LPS synthesis reduce the number of surface LamB proteins [35] while it has also been suggested that altered LPS configuration leads to non-optimal arrangement of LamB receptors, by analogy to the T4 phage receptor OmpC [36], [37]. These explanations are not mutually exclusive.

The second group of *E. coli* genes identified in our screen consisted of 34 genes with known functional roles but no previous link to lambda phage infection. Interestingly, the largest functional category in this group appears to be metabolic. Several of the identified genes play a role in central metabolism, including several key enzymes (*pgi*, *pgm*, *atpA*, *talB*, *fucA*), transporters (*crr*, *lamB*, *manZ*), and regulators (*cyaA*, *malT*, *malI*, *fruR*, *bglG*, see Figure 2C). Furthermore, the glucokinase encoded by *glk* was not included in our initial list but likely inhibits lambda replication as only one relatively small plaque was observed in our screen.

*iscS*, *tusA*, *tusE*, and *mnmA* were all found in our initial screen and are involved in the thiolation of tRNA [38]. *iscS* is necessary for thiolation of all nucleosides while *tusA*, *tusE*, and *mnmA* gene products contribute specifically to 5-(carboxy)-methylaminomethyl-2-thiouridine modification of tRNA^Glu^, tRNA^Gln^, and tRNA^Lys^.

Several other interesting genes emerged in the second group. *spr* is an outer membrane lipoprotein, which for certain mutants, show thermosensitivity [39]. Intriguingly, mutants of *nlpI*, also found in our screen, have been shown to suppress this sensitivity [40]. *pepA* expresses aminopetidase A/I, which is involved in gene regulation, maintaining plasmid monomers, and preventing plasmid trans-recombination [41]. RlmE is the methyltransferase responsible for methylating U2552 of 23S rRNA [42]. Mutants for *rlmE* show reduced growth rate, protein synthesis activity, [43] and can modulate translational accuracy [44].

Four genes found using the screen are essentially uncharacterized. The structure of YbeD has been determined and may play a role in lipoic acid biosynthesis or the glycine cleavage system [45]. Global bioinformatic analysis revealed that YneJ has a LysR-type DNA binding domain and may therefore be a transcription regulator [46]. YfiM might act as an ABC transporter [47] and *yecR* has appeared in a computational screen to identify genes regulated by the flagellar master regulator FlhD2C2 [48].

### Infection dynamics of *E. coli* strains showing reduced infectivity

We wondered how best to characterize the functional roles of new genes implicated in lambda infection. One weakness of the previous screens for host gene requirements in viral infection is that the information produced by these screening experiments is highly limited, generally involving only a few measurements per gene.

**Figure 3:**
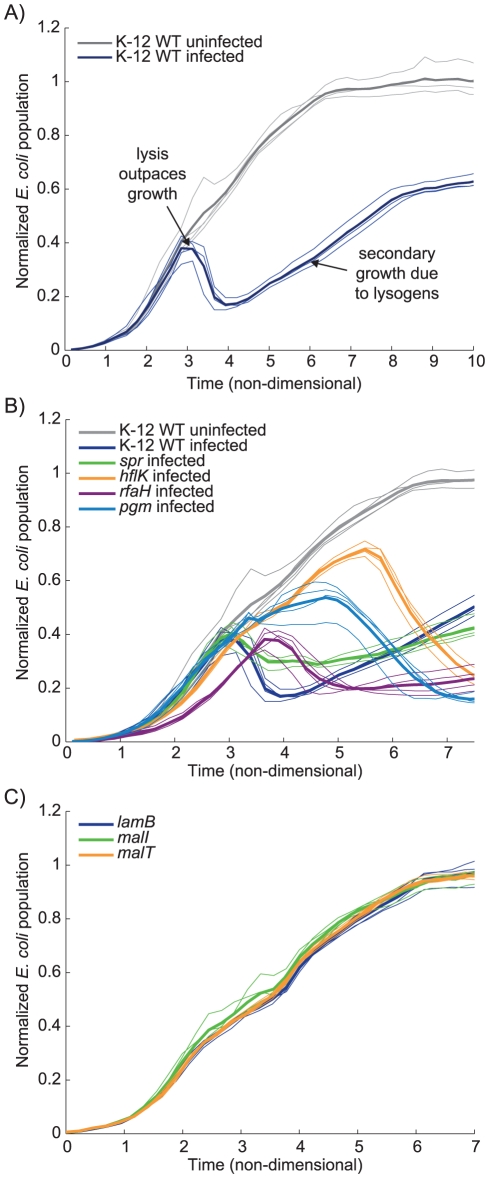
*E. coli* growth curves under normal conditions and incubated with lambda phage. All four replicates are shown with the average displayed as a bolded line. These growth curves have been normalized by growth rate and maximum growth capacity to facilitate comparison between strains (see Materials and Methods). (A) A typical set of K-12 WT uninfected (gray) and infected (blue) growth curves. (B) A sample of infected knockout strain growth curves to demonstrate the variability between strains, with respect to the response to infection. (C) The Δ*lamB*, Δ*malI*, and Δ*malT* strain growth curves, which appear in the same pathway (see Figure 2A), also exhibit very similar infected growth dynamics.

We hypothesized that higher time-resolution monitoring of infection dynamics would facilitate validation and further characterization of the roles these host genes play in the lambda phage lifecycle. We monitored *E. coli* growth and lysis over a full course of lambda infection in liquid culture (see Figure 3A). Infected K-12 WT bacteria grow exponentially for about 3 hours, after which the rate of bacterial lysis briefly outpaces growth. During this time, many phage have also induced lysogeny in their *E. coli* hosts, which then become resistant to further lytic infection. The lysogenic strains eventually take over the culture, growing exponentially until stationary phase.

We monitored growth of infected and uninfected cultures for all 57 strains that showed reduced infectivity in our plaque assay. We found that the infection dynamics varied significantly between strains. Figure 3B shows selected time course data, normalized by growth rate and maximum carrying capacity (i.e., optical density at stationary phase) to highlight the difference between infected and uninfected strains (The infection time courses for all 57 strains can be found in Figure S1).

We observed that genes with directly cooperative roles in lambda infection often exhibited very similar growth and clearance dynamics. As a simple example, Figure 3C shows the infection time courses for Δ*lamB*, Δ*malI*, and Δ*malT*. As mentioned above, all of these genes work together—*malT* and *malI* regulate the expression of the *lamB* transcript and functional protein product.

### Clustering of infection dynamics

**Figure 4:**
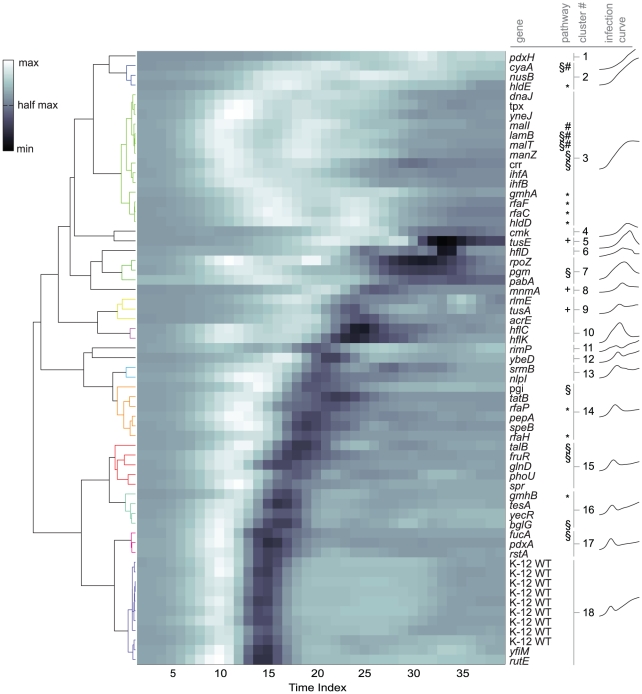
Clustered *E. coli* growth curves under infection conditions for all of the reduced infectivity strains. Each row of the heatmap is the derivative of an averaged, normalized, and smoothed time course for a single strain (see Materials and Methods). A dendrogram indicating the relative distance between row is shown at left. At right and proximal to the clustergram, the corresponding gene names are listed, followed by the cluster number for significant groups. Also shown are symbols indicating genes that fall into either the *lamB* regulation pathway (#, see Figure 2A), the LPS inner core biosynthesis pathway (*, see Figure 2B), central carbon metabolism and regulation (§, see Figure 2C), and tRNA thiolation (+, see Figure 9). At the far right are averaged time courses for each of the significant clusters.

This observation suggested that similar infection time courses between knockout strains might be an indicator that the corresponding gene products act together in a pathway required for lambda infection. We performed agglomerative hierarchical clustering on the processed time course data to help identify knockout stains with similar infection dynamics (see Figure 4). To reduce the effects of varying growth rate between strains and to focus on the key transitions in the infection time courses, we pre-processed our data to obtain a normalized time course of the infection curve derivative for each strain (see Materials and Methods).

In all, we identified 18 separate clusters, separated primarily by: (i) the time point at which cell lysis outpaced cell growth, (ii) the length of time to achieve maximum clearance, and (iii) the re-growth or lysogenic growth rate. As an example, Clusters 17 and 18 show similar dynamics for properties (i) and (ii), but Cluster 17 shows limited secondary/lysogen growth. Genes in clusters with limited secondary growth are particularly interesting because they may play a role in lysogenization, as exemplified by Cluster 9, which contains *hflC* and *hflK*—two known regulators of lysogenization. However, this interpretation comes with the caveat that the secondary growth occurs under slightly different nutrient conditions than the primary growth phase.

Two major clusters bookended the dataset. At one end, Cluster 18, which was largely comprised of K-12 WT samples, showed rapid growth and lysis followed by robust secondary growth. Interestingly, two genes, *yfiM* and *rutE*, which were identified in our initial screen and consistently showed small plaques in the plaque assay, clustered with this group.

The largest cluster, Cluster 3, falls at the opposite end of the spectrum from the K-12 WT cluster and is characterized by little or no lysis over the entire time course. This cluster includes many of the previously known *E. coli* genes whose absence prevents lambda infection, such as *lamB*. There is a strong correlation between the plaque assay and infection time course as eight (Δ*dnaJ*, Δ*yneJ*, Δ*malI*, Δ*malT*, Δ*lamB*, Δ*manZ*, Δ*rfaF*, and Δ*rfaC*) of the nine strains that had zero plaques in all trials fell into this cluster (see Table S1). The one exception corresponds to the outer membrane lipoprotein named *spr* (Cluster 15). Of the remaining genes in this cluster, Δ*tpx*, Δ*ihfB*, Δ*gmhA*, and Δ*hdlD* showed zero plaques in the initial screen and small plaques in the validation plaque assays. The two remaining genes in this cluster, Δ*crr* and Δ*ihfA*, produced small plaques in each plaque assay and very little clearance in the liquid culture assay.

Several other genes involved in the same biological pathway were also found in the same cluster, for example, *hflC* and *hflK* (Cluster 10); *lamB*, *malT*, and *malI* (Cluster 3); and many of the LPS pathway genes (Cluster 3). Interestingly, *pgi* and *talB* (Clusters 14 and 15) both catalyze formation of fructose-6-phosphate and demonstrate similar infection dynamics. However, some genes involved in shared pathways did not cluster together. For example, Δ*pdxH* and Δ*pdxA* showed very different dynamics and clustered at opposite ends of the clustergram.

**Figure 5:**
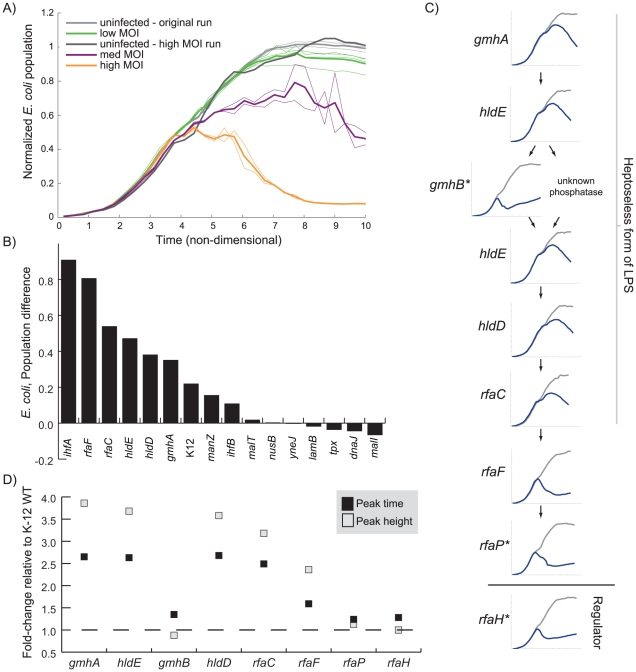
The effect of increased phage concentration on infection dynamics. All of the strains in Cluster 2 and 3 (except *ΔcyaA* and *Δcrr*) were tested at three MOIs; the results for the Δ*ihfA* strain are shown in (A) and the rest are found in Figure S2. The bold line indicates the average value of all four measurements. (B) The difference in normalized *E. coli* concentration between the high and low MOI tests at eight normalized time units is plotted for each strain (The Δ*nusB* strain time course progressed to only 7 normalized time units due to slow growth but exhibited little difference between MOI tests at that time). (C) For the LPS inner core biosynthesis strains, the high MOI infection growth curves (uninfected in gray and infected in blue) are shown in order of pathway occurrence (top to bottom) to highlight how the dynamics change depending on pathway position. MOI results for strains highlighted with an asterisk were not tested at the higher MOIs and therefore the low MOI results are shown. (D) The time (peak time) and absorbance (peak height) where clearance begins to be detected for each of the time courses shown in (C) is plotted relative to K-12 WT.

The finding that strains from multiple pathways all exhibited similar dynamics in Cluster 3 was intriguing, and we wondered if we could further discriminate between members of this cluster. We monitored the total *E. coli* concentration during infection at higher (10- and 100-fold) multiplicity of infection (MOI) for each strain in the cluster (see Figure 5A and Figure S2). After normalization, we compared the difference in the population between the uninfected and infected samples (see Figure 5B). We found that the higher MOI values appeared to have no effect on several strains, including Δ*lamB*, Δ*dnaJ*, Δ*malI*, Δ*malT*, Δ*nusB*, Δ*tpx*, and Δ*yneJ*. *tpx* and *yneJ* are particularly interesting as their roles in lambda phage infection are entirely unknown. Those strains that did appear to be affected by higher MOIs corresponded to the genes involved in biosynthesis of inner core LPS, *ihfA*, *ihfB*, and *manZ*.

At the higher MOIs, the genes involved in LPS synthesis appeared to separate into two groups based on peak times (see Figure 5C and 5D). With the exception of *gmhB*, the genes required for attaching heptose to the KDO group had nearly identical peak times. Others have shown that Δ*gmhB* does not produce an entirely heptoseless form of LPS and conclude that there is another phosphatase that can catalyze this reaction [49]. Δ*rfaF*, Δ*rfaP*, and Δ*rfaH* had peak times much closer to K-12 WT.

### Computation model of infection dynamics and the characterization of *yneJ*

We found the identification of unannotated gene *yneJ* to be of particular interest. Infected Δ*yneJ* showed no visible plaques and growth dynamics nearly identical to uninfected samples. We decided to apply mathematical modeling to interpret our infection time courses and help direct our efforts in characterizing the role of *yneJ* in lambda phage replication.

**Figure 6:**
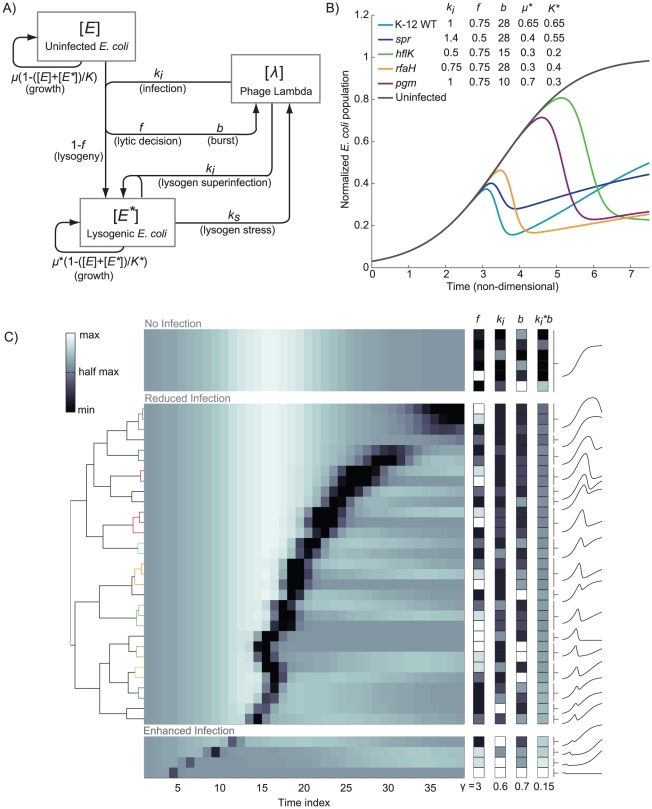
Numerical simulations of *E. coli* growth during phage lambda infection. (A) Schematic of the mathematical model, where the boxes represent the amounts of uninfected ([*E*]) and lysogenically-infected ([E*]) *E. coli* as well as phage ([*l*]). The arrows indicate the effects of one variable on the others, and are labeled with the relevant parameters. A detailed description of the model is given in Materials and Methods. (B) Simulated infection time courses where the model parameters were varied (inset) to produce trajectories that closely resembled the data shown in Figure 3B. (C) Clustergram of simulated time course derivatives for a variety of parameter combinations. 125 simulations were generated using the model (combinations of five possible values for each key parameter, see Figure S3 and Figure S4). Of these, the derivatives of all of the simulation time courses with low infectivity were clustered (middle), using the same technique as with the experimental data shown in Figure 4. Additionally, six of the simulations that exhibited no infectivity, and four of the simulations that exhibited high infectivity are shown at top and bottom as representative examples. Similar to Figure 4, a dendrogram is shown at left, and cluster indicators and growth curves representing the average behavior of each cluster are shown at right. At right and proximal to the clustergram are columns indicating the relative values of model parameters *f*, *b* and *k_i_*, as well as the product of *k_i_* and *b*, adjusted for display on the same color mapping by standard gamma correction of value shown.

The population level interaction of phage with bacterial hosts has previously been phrased as a predator-prey system of differential equations [50], [51]. Following these previous efforts, we constructed a model that defines three populations as concentrations: uninfected bacteria, lysogens, and infectious phage (see Figure 6A). We then considered the effects of three key parameters on infection dynamics: (i) the burst size *b*, meaning the average number of infectious phage released upon host cell lysis, (ii) the fraction *f* of infection events that proceed down the lytic pathway, and (iii) the rate *k_i_* at which infection occurs. We found that we could recapitulate the infection time courses we observed simply by varying the parameters in our model (see Figure 6B, compare to Figure 3B).

To determine how model parameters could create different phenotypes, we simulated an infection time course for five levels of each of these three key parameters (125 simulations in total). The parameter combinations led to simulations that strongly resemble virtually all of the experimental time courses we observed (see Figure S3). We clustered the derivatives of the simulated time courses and found that the variation between simulations likewise resembled the variation between experimental strains (see Figure 6C and Figure S4). The one significant exception to this observation was that many of the simulations actually led to enhanced phage infection.

Importantly, the same types of variation that were found in the experimental time course cluster were also found in the simulated data cluster. We therefore wanted to determine which parameter combinations contributed to which type of variation. We found that the variation in *f* primarily contributes to the re-growth of the lysogenic population (see Figure 6C, right). In contrast, there is some correlation between *b* and *k_i_* (see Materials and Methods), but we found that the product b*k_i_ varies inversely with the time at which lysis outpaces cell growth (see Figure 6C, far right).

**Figure 7:**
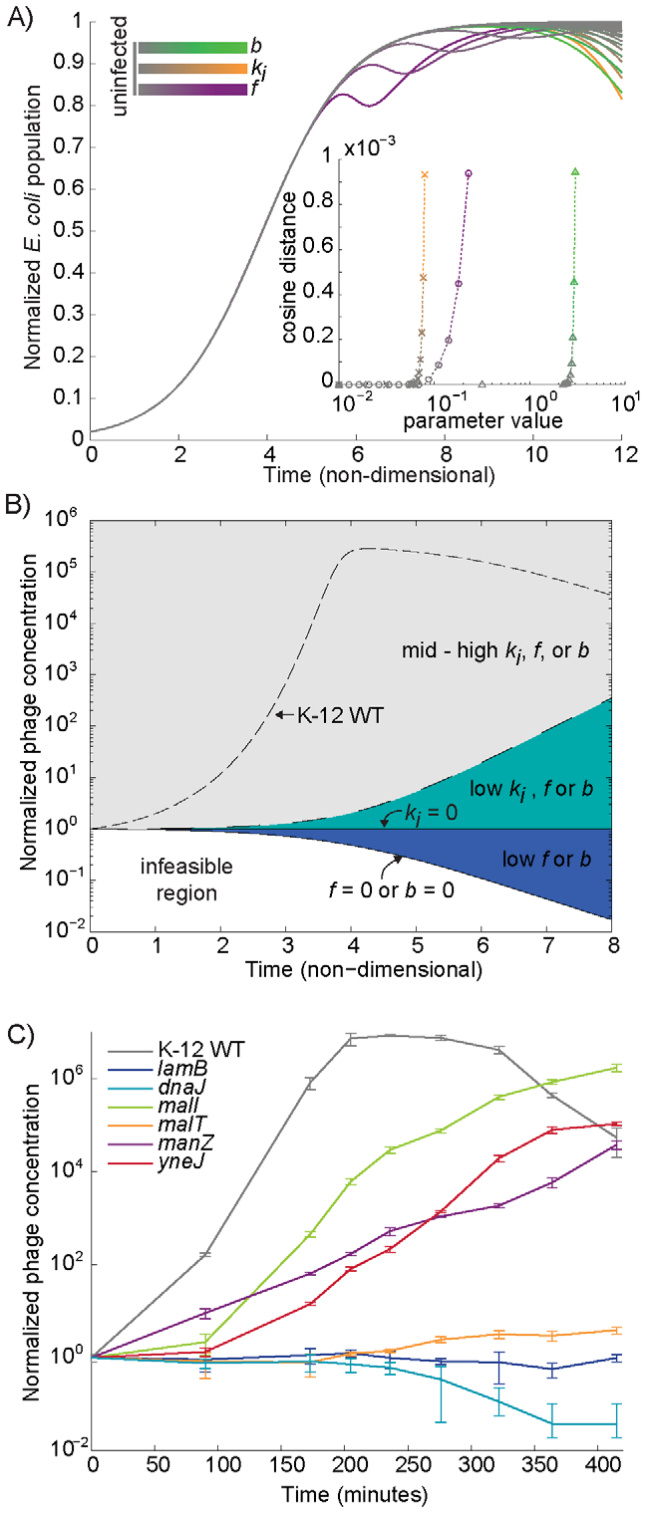
The use of phage production time courses to further discriminate between strains with similar *E. coli* infection curves. (A) Certain combinations of model parameters produce indistinguishable simulated infection curve phenotypes. Some simulated time courses are shown. Inset quantifies the eventual deviation, as cosine of angle included between simulated time course vectors, with the variation of each parameter away from zero while other two parameters at constant *k_i_* = 1, *b* = 28, *f* = 0.75. (B) A phase plane-like diagram shows how the simulations shown in (A), although identical in terms of *E. coli* growth and lysis, are often different in terms of phage production. The regions in the diagram are labeled by the particular parameter combinations that simulate phage production in the region. The dashed line separating the gray and teal regions indicates a soft threshold where we considered dynamics significantly deviating from uninfected curves. (C) Experimentally measured phage production time courses for several of the Cluster 3 strains, shown as fold change from infection phage concentration.

This relationship between the parameters *b* and *k_i_* undermines the hypothesis that strains with a similar infection time course are involved in the same pathway, because multiple parameter combinations can lead to identical time courses. For example, the no-infection phenotype exhibited by members of the *lamB* cluster can be created computationally by setting *f*, *b*, or *k_i_* to very low values (see Figure 7A for the simulations and Figure 3C for representative data). However, the computational scenarios that produce equivalent total *E. coli* concentration time courses (see Figure 7A) create significantly more variation in the lambda phage concentration time courses (see Figure 7B). Specifically, the situation when lambda cannot infect the host at all (equivalent to setting *k_i_* to zero) leads to a stable concentration of phage over time, and when no viable phage are produced by infection (a low or zero value for *b*), the phage concentration is steadily reduced over time. In the third scenario, viable phage is produced at a rate exceeding its absorption (initially) and thus accumulates, however rate of production is very low (low *f*, *k_i_*, or *b*) and insufficient to produce host lysis on an observable scale. Accumulated phage populations in the latter case are orders of magnitude lower than observed for wild type.

To test these model predictions, we compared them to phage concentration time courses that we generated for members of the *lamB* cluster (see Figure 7C). We found that the simulated time courses compared favorably to our experimental data, in that infection of the *lamB* strain led to a nearly constant phage concentration, as would be expected if phage coul;d not infect the host. We also found that infection of the *dnaJ* strain, which is necessary for replication of lambda DNA, led to a decrease in phage concentration. In contrast, the Δ*manZ*, Δ*malI*, and Δ*yneJ* strains all produced significant amounts of phage over time, albeit less than the K-12 WT.

**Figure 8:**
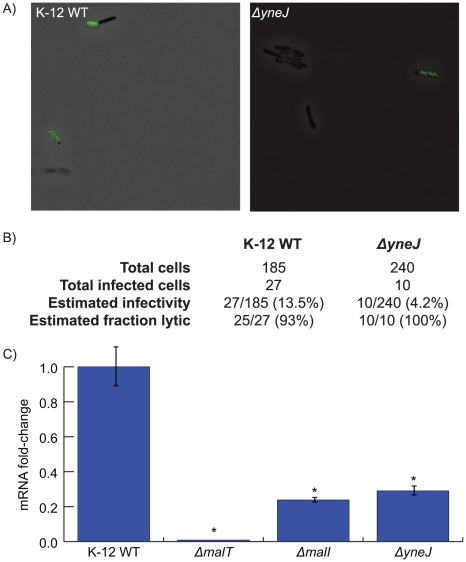
Determining the function of *yneJ*. Single cell analysis of *E. coli* infection in the presence of GFP expressing lambda phage was performed to assess *yneJ*'s effect on infection rate and the lytic-lysogenic decision. Growth of the infected K-12 WT and *ΔyneJ* strains were observed at 60× magnification and assessed for GFP expression. (A) Images of K-12 WT and *ΔyneJ* cells infected with GFP expressing phage. A recently lysed cell can be seen in the bottom left corner of the K-12 WT image. (B) Table summary of data obtained from single cell analysis shows reduced infectivity and no significant change in fraction lytic for *ΔyneJ*. (C) Bar plot showing the results of quantitative real-time RT-PCR of *lamB* mRNA, for *ΔyneJ* as well as several cell lines deficient in known *lamB* transcriptional regulators. The error bars indicate the standard deviation. The asterisk indicates a fold-change between the K-12 WT and the strain of interest with a p-value ≤0.001.

Our results of the phage concentration time courses suggest that the effect of Δ*yneJ* on lambda infection is within the low *f*, *k_i_*, or *b* range. To investigate if the loss of *yneJ* might affect the lytic-lysogenic decision (*f*) or the relative infection rate (*k_i_*), we performed single cell analysis of K-12 WT and Δ*yneJ* cultures infected by GFP-expressing lambda phage (see Figure 8A). We observed a large decrease in the percentage of Δ*yneJ* cells initially infected relative to K-12 WT (see Figure 8B), while lysis occurred at approximately the same time relative to K-12 WT. In addition, K-12 WT demonstrated a very low frequency of lysogeny, where only 2 of 27 infected cells appeared to become lysogens. We did not observe any lysogens among the 10 infected Δ*yneJ* cells. Based on these results, it does not appear that the reduced infectivity of lambda phage in Δ*yneJ* is due to a high frequency of lysogens (low *f*), but more likely, a reduced infection rate (low *k_i_*). This is consistent with Δ*yneJ* showing similar dynamics to other knockouts that are known to affect infection rate (*i.e.*, Δ*manZ*, Δ*malI*, and Δ*malT*) for both the infection and phage concentration time courses.

We hypothesized that similar to MalI and MalT, YneJ might play a role in the regulation of *lamB*. To test this hypothesis we used quantitative real-time RT-PCR to examine *lamB* mRNA expression in K-12 WT, *ΔlamB*, *ΔmalT*, *ΔmalI*, and *ΔyneJ* cultures. We found that *ΔyneJ* had a significantly reduced expression of *lamB* (see Figure 8C). *lamB* mRNA levels in *ΔyneJ* were comparable to levels found in *ΔmalI* mutants.

### U34 tRNA thiolation and lambda infectivity

Sulfur used for the thiolation of tRNA nucleosides in *E. coli* is first transferred to IscS. Depending on the tRNA and the particular base modification there are two distinct pathway classes: an iron-sulfur ([Fe-S]) protein-independent and a [Fe-S] protein-dependent pathway [52]. Our plaque assay results found several genes in the [Fe-S] independent pathway responsible for thiolation of U34 for tRNA^Glu^, tRNA^Gln^, and tRNA^Lys^. In this pathway, sulfur is transferred from IscS to TusA. TusB, TusC, and TusD form a heterotrimer complex where Cys78 of TusD is able to form persulfide, facilitating transfer of sulfur from TusA to TusE [38]. Sulfur is then passed from TusE to MnmA, which thiolates uridine 34 of the tRNA. Our plaque assay screen showed reduced infectivity for all strains in this pathway with the exception of Δ*tusB*, Δ*tusC*, *and* Δ*tusD*. For Δ*tusB*, Δ*tusC*, *and* Δ*tusD*, we do observe a slight reduction in efficiency of plating and the plaques are generally smaller then the K-12 WT controls (data not shown).

In the [Fe-S] dependent pathway, sulfur is transferred from IscS to IscU. This transfer is facilitated by several proteins, including IscA, Fdx, and the chaperone/co-chaperone pair HscA and HscB. As it is thought that IscU binds to IscS, potentially competing with TusA for sulfur, we looked more closely at the proteins in the [Fe-S] dependent pathway. We found that Δ*iscU*, Δ*hscA*, and Δ*hscB* showed significantly larger plaques compared to K-12 WT (data not shown).

**Figure 9:**
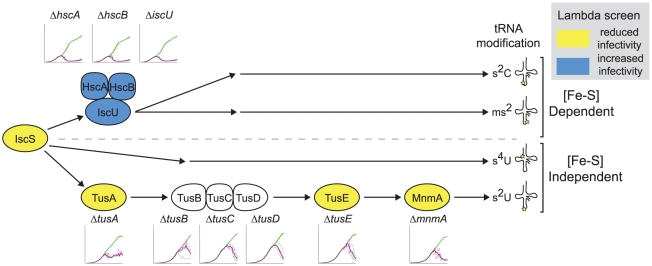
Pathways involved in sulfur metabolism and the thiolation of tRNA nucleosides affect lambda replication both positively and negatively. Several genes identified in this study fall within tRNA thiolation pathways, as shown schematically here. A star on the secondary structure drawing at right indicates the tRNA modification location. Infection dynamics are displayed (Absorbance (600 nm) vs. Time (hours). The scaling is equivalent for all plots) for knockout strains, demonstrating consistent dynamics within pathways. The pathway that includes *ΔtusA*, *ΔtusBCD*, *ΔtusE*, and *ΔmnmA* shows dynamics consistent with decreased infectivity (yellow), while the iron-sulfur dependent pathway that goes through IscU shows dynamics consistent with increased infectivity (blue). *ΔiscS* has a very slow growth rate and is therefore not shown here.

In addition to the four tRNA genes identified in the original screen, we examined the infection dynamics for Δ*tusB*, Δ*tusC*, Δ*tusD*, Δ*iscU*, Δ*hscA*, *ΔhscB* and a *ΔtusBCD* strain (see Figure 9 and Figure S5). The Δ*tusB*, Δ*tusC*, and Δ*tusD* strain infection dynamics were indicative of reduced infectivity, showing a significant delay in the peak. *ΔtusBCD* had very similar dynamics to the individual knockouts for *tusB*, *tusC*, and *tusD*. The infection dynamics for Δ*iscU*, Δ*hscA*, and *ΔhscB* cleared relatively quickly and had very little secondary growth.

## Discussion

In summary, to determine the *E. coli* gene requirements for lambda infection, we performed a screen of 3,985 non-essential gene knockout strains and found 57 strains with impaired lambda infectivity. In addition to identifying many genes with established roles in lambda phage infection, we found a surprising number of previously unassociated genes, four of which are currently unannotated. In addition, several of the genes found in our screen have human orthologues (see Table S2).

Central metabolism was the largest shared functional category among our results. In particular, we found many genes involved in multiple entry points to and regulators of glycolysis. This observation is in part related to lambda phage's dependence on LamB and the factors that regulate *lamB* expression. Several other metabolic genes identified in our screen are known to play a role in regulating the lytic-lysogenic decision. Based on this observation, it is tempting to speculate that many of these genes may influence lysogeny, whether by sensing or altering the cell's nutritional state. Our results potentially expand this lytic-lysogenic network and provide a framework for a deeper understanding of this decision as well as the metabolic requirements for lambda phage replication. It is interesting to note that many of the host-dependencies identified in the recent mammalian virus screens are also involved in energy metabolism and enzymes involved in amino acid and nucleic acid synthesis.

Second, we demonstrated that the knockout strains for genes with common roles in infection often showed similar infection dynamics. Some examples include the *hfl*, *ihf*, LPS biosynthesis and *lamB*-related genes. Our observation is complicated by at least three factors. One complication arises when the effect of gene deletion within a pathway varies depending on the step within the pathway, as in the LPS biosynthesis pathway. Another confounding factor is that some proteins may be partially redundant, where in the absence of one protein another can adequately compensate thus producing a mild or no change in phenotype. This may be the case for GmhB and an unidentified phosphatase. While we did not identify the complementary phosphatase in this study, one might look among the strains clustering with *ΔgmhB*. Finally, many proteins have pleiotropic effects and the observed dynamics may arise from the modulation of multiple pathways. An example of this is the protein CyaA, which has a metabolic function but also plays a role in the lytic-lysogenic decision. Notwithstanding these exceptions, dynamic infection data was extremely useful as a validation tool and a “first-step” in assessing host gene functionality in lambda infection.

We used a combination of computational modeling and further experimentation to characterize many of the strains in more detail. This was best exemplified by our efforts to characterize the previously unannotated gene, *yneJ*, and its apparent critical role in infection. We observed that Δ*yneJ* closely resembled the Δ*manZ*, Δ*malT*, and Δ*malI* strains in terms of growth phenotype, lambda phage production, and response to higher phage concentration. Using single-cell imaging of GFP expressing lambda phage, we found that Δ*yneJ* is not immune to infection and does not appear to regulate the lytic-lysogenic decision. We suspected that *yneJ* may play a role in attachment or entry, possibly through the regulation of LamB or another unidentified membrane-associated protein and tested the expression levels of *lamB* in several strains including Δ*yneJ*. Our data demonstrated a reduced level of *lamB* mRNA in Δ*yneJ* similar in magnitude to Δ*malI*. Given YneJ's LysR-type DNA binding domain, we speculate that YneJ is an upstream regulator of *lamB* transcription—possibly directly or indirectly regulating *malI* expression. Given its effect, it is intriguing that *yneJ* was not identified over the several decades of reverse-genetic screening of phage lambda.

Another surprising result of this work was the identification of two important pathways for lambda phage replication with a common node at cysteine desulferase IscS. Interestingly, inactivation of one of these pathways (including *tusA*, *tusB*, *tusC*, *tusD*, *tusE*, and *mnmA*) leads to impaired lambda infection, while inactivation of the other pathway (including *iscU*, *hscA*, and *hscB*) facilitates lambda infection.

Others have shown that the 5-(carboxy)-methylaminomethyl-2-thiouridine modification at the tRNA wobble position increases frame maintenance and prevents codon-specific frameshift in *E. coli* [53]. We hypothesize that the rapid and proper synthesis of lambda phage proteins is particularly dependant on this modified nucleoside. Viruses of infected cells lacking this modification likely show decreased production of functional viral proteins and thus produce a smaller number of infectious particles. In concept, this hypothesis resembles the lethal mutagenesis therapeutic strategy for treating viral infection [54], albeit at the protein level. Based on our data, it appears that 2-thiouridine modifications of tRNA^Glu^, tRNA^Gln^ and tRNA^Lys^ are particularly important for the efficient production of infectious lambda phage particles. The s^2^U34 modification is conserved for several tRNAs across all organisms [55], raising the question of whether these genes or related pathways might present novel anti-viral targets for mammalian viruses.

## Materials and Methods

### “Keio Collection” screen

Our screen was performed using the established plaque assay method [56]. K-12 WT (ATCC, 47076) and all strains of the “Keio Collection” were cultured overnight (∼16 hours) in nutrient broth (NB, 8 g/L, Fluka Analytical, N7519) using 96 deep-well plates (Nunc, #278743). Incubation was done at 37°C while rotating at 225 RPM. Each *E. coli* strain was incubated with bacteriophage lambda (ATCC, 23724-B2) for 30 minutes at 37°C—optimal titers for bacteriophage lambda were determined beforehand using a dilution series. 2% 2,3,5-Triphenyltetra-zolium chloride (Sigma, T8877) was added to NB top agar (0.04% final concentration) and incubated at 55°C for 30 minutes. The top agar was then added to the incubating *E. coli* and phage samples and 80 µl was plated onto 24-well agar plates. The agar plates were made the previous day with 500 µl of NB bottom agar for each well. Included on each plate were two replicates of infected K-12 WT and one well of *ΔlamB*. After 24 hours, the number of plaques generated by infection of each strain was counted and compared to K-12 WT counts. Wells with significantly fewer or smaller plaques for two replicate experiments were considered “hits”.

### Infection time courses

To monitor the *E. coli* concentration of an infected batch culture over time, we used an incubated plate reader (Perkin-Elmer Victor3, 2030-0030). Strains of the “Keio Collection” were inoculated and grown overnight (∼16 hours) in 2 ml NB in 5 ml round-bottom tubes. The next morning the samples were diluted 1∶100 in fresh NB media and grown for 3 hours. After 3 hours the samples were measured for absorbance at 600 nm and diluted in NB to 0.1 OD. 15 µl of 0.1 OD *E. coli* and 15 µl of ∼10^4^ plaque forming units/ml (pfu/ml) lambda stock were added to 170 µl of NB in 96-well plates—representing an MOI of ∼2×10^−4^ pfu/bacteria. Four replicates for each low infectivity strain (infected and uninfected) were assayed. Strains *Δatp*A, *ΔthyA*, and *ΔiscS* did not show significant growth rates over the time course, and therefore provided no additional information concerning phage infection. The “Keio Collection” was created using *E. coli* BW25113 as the background strain, so we compared the infection dynamics of this strain with *E. coli* MG1655 and found them to be essentially identical (see Figure S6).

Included on each plate were replicates of K-12 WT. The incubation protocol included an initial 10 minute shake (double orbital, 1.5 mm diameter, normal speed), followed by 38 cycles consisting of the following actions: a one second absorbance measurement at 600nm (Perkin Elmer, 600/8nm, 1420-521), 5 µl injection of milliQ water into each well (to counter volume loss due to evaporation), and a 10 minute shake (double orbital, 1.5 mm diameter, normal speed). The time course was performed at 37°C for approximately eleven hours.

### Lambda phage time course quantification

The K-12 WT, Δ*lamB*, Δ*manZ*, Δ*malT*, Δ*malI*, Δ*dnaJ*, and Δ*yneJ* strains were inoculated in NB and grown overnight at 37°C while rotating at 225 RPM. The next morning the samples were diluted 1∶100 in fresh NB media and grown for 3 hours. After 3 hours the samples were measured for absorbance at 600 nm and diluted in NB to 0.1 OD600. 375 µl of *E. coli*, 375 µl phage stock, and 4.25 ml of NB at 37°C were combined in 14 ml tubes. Aliquots were taken from each culture at 90, 173, 205, 236, 276, 322, 364, and 415 minutes post-infection and filtered using 0.2 µm PES filters (Nalgene, 180-1320). The filtrate was then diluted in SM + gelatin (0.058% (w/v) NaCl, 0.02% (w/v) MgSO4-7H2O, 50 mM Tris (pH 7.5), 0.01% gelatin). 100 µl of these dilutions were incubated with an equal quantity 2.0 OD600 *E. coli* in 10 mM MgSO_4_ for 10 minutes before combining with 1 ml NB top agar 500 µl onto 6-well plates. After incubating overnight, the plaques were counted to determine the pfu/ml at each time point.

### Infection time course data processing

A key challenge in our data processing was to determine a metric to compare two infection time courses. Observing that uninfected knockout strain time courses cluster by pathway based solely on the variation in background growth phenotype, normalization of infected growth curves was necessary in order to avoid clustering these based on the similar behavior of their bounding uninfected curves. To prevent clustering based on growth rate and the eventual population limit, we non-dimensionalized the growth time courses for each strain using parameters of logistic population growth fit to uninfected data (model presented, simulated as uninfected using initial condition [*λ*] = 0 at t = 0). The carrying capacity, *K*, was considered the characteristic population and inverse growth rate 1/*μ* the characteristic time. This treatment allowed us to compare the key features of the infected time courses relative to their background, for example allowing distinction between a strain that is overtaken by phage late due to generally weak/slower growth/metabolic function and one that is overtaken late despite quick/normal growth potentially indicating a more phage specific knockout impact. Although deemed necessary for the meaningful data interpretation via our method, anytime rescaling is performed to clarify certain dataset features, the potential exists to deemphasize or obscure other features of that data set, and we have thus presented our entire raw dataset in Figure S1. Data was smoothed using the robust lowess implementation in MatLab (window size 0.1 as a fraction of total data points), followed by analytical calculation of the time derivative from a cubic smoothing spline to avoid noise amplification. A cosine distance metric was used in clustering, 1-*θ*, where theta is the included angle between time course derivatives treated as vectors, emphasizing similarities in population change direction rather than magnitude. Agglomerative hierarchical cluster tree was constructed with average linkage, and clusters defined by a linkage of less than 0.16.

### Computational modeling of lambda phage infection dynamics

We constructed a model that defines three populations as concentrations: uninfected bacteria [*E*], lysogenically-infected bacteria [E*], and infectious phage [*λ*]. Bacterial growth is assumed to occur logistically with rate *μ* towards a limiting population [*K*]. We used analogous but separate parameters for lysogens (denoted by *) [57].

The ordinary differential equations are as follows:
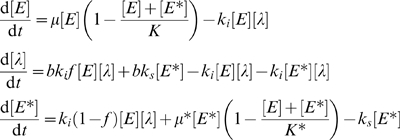
Mass-action kinetic infection of bacteria by phage, with rate *k_i_*, is well established [58]. Superinfection of lysogens occurs with no effect other than to reduce the infection phage population [59]. The amplification factor parameter *b* represents the average number of infectious phage released upon host cell lysis. Lysogen induction to the lytic pathway by DNA damaging stresses is included at rate *k_s_* for the sake of completeness, but is assumed negligible under our conditions and henceforth neglected. *f* is the fraction of infection events that proceed down the lytic pathway. Given the complex nature of the lambda decision and switch (and neglect of *k_s_*), *f* as a constant fraction is necessarily an average parameter containing information on the ease of lysogeny establishment and maintenance.

For the purposes of well-scaled numerical simulation the model was converted to a non-dimensional form using the same characteristic time and population as for the experimental time courses. Replacing the time with its product with *μ*, and dividing each population variable by *K*, both maximum growth rate and maximum population reduce to unity. The parameters *f* and *b* are retained as fraction and amplification factor respectively, while lysogen growth reduce to ratios to their analogous uninfected growth parameters, and *k_i_* becomes the dimensionless group *k_i_K*/*μ*. All simulations presented were completed using the scaled form, and values of parameters listed are the corresponding non-dimensional form or group.

All numerical integration was completed using the standard MatLab implementation of rk45, with default tolerances. The initial condition [*E*]_t = 0_ = 0.02 was chosen and used for all simulations based on values fit simultaneously as logistic kinetics were fit to experimental data (least squares distance), with [*λ*]_t = 0_ set using the experimentally determined MOI, and no initial lysogen population ([E*]_t = 0_ = 0). The simulation results for the *E. coli* population presented are the sum [*E*]+[E*], as these populations are not distinguished experimentally during our infection time course observation. Simulations were clustered using the same method as experimental data (though smoothing was unnecessary). Parameter values used in the simulations, displayed in Figure S3 and Figure S4, were all possible combinations of *k_i_* = {0, 0.25, 0.75, 2, 5}, *f* = {0, 0.5, 0.75, 0.95, 1}, *b* = {0, 10, 20, 50, 100}. Values for *f* and *b* were chosen as physically reasonable values. The set of values for *k_i_* were then chosen from the range that produced model behavior resembling our experimental observations. It should be noted that for a given infection time course simulation, a practically equivalent bacterial population time course can be generated using alternate combinations of the parameter *k_i_* and *b* values. The lysogen growth character was not varied, using constant μ* = K* = 0.7 for all simulations, both because our primary focus was on the character of the infection process that leads to the initial overtake of the bacterial population by phage, and also because at later times in batch culture it is possible that our experimental observations of lysogen growth character are dominated by nutrient limitation.

### Single-cell imaging of GFP–expressing phage infection

Exponentially growing cells were infected with lambda b::GFP kanR [17]. Adsorption of lambda (MOI≈0.5) was done at 4°C for approximately 20 minutes. 1 µl of sample was pipetted onto a 2% agarose pad. The agarose pad was made using equal volumes of 4% LMP agarose and 2× EZ-RDM (Tecknova, M2105) supplemented with 2% maltose, 20 mM MgSO_4_, and 1mM of IPTG to induce expression of the GFP construct. The pad was then inverted onto a cover slide and a plastic lid was placed on top to help prevent drying of the pad over the time course. The assembled slide was maintained at 37C during imaging. Both phase (30 ms) and GFP (50 ms) images were taken every 2 minutes at 60× magnification. Cells were manually assessed for infection.

### Quantitative real-time RT–PCR of *lamB*

Exponentially growing cells were treated with RNALater (Ambion, AM7020) according to the manufacturer's recommendations. The RNA was then isolated using Qiagen's RNeasy Mini Extraction kit (Qiagen, 74104). DNase digestion was performed on the RNA sample using Deoxyribonuclease I (Invitrogen, 18068-015). First-strand cDNA synthesis was performed (Invitrogen, 18080-051) followed by RNase H digestion. 100 ng of template cDNA and primers (200 nM final concentration) were combined with the SYBR Green PCR master mix (Applied Biosystems, 4367659). *LamB* (left primer, 5′- ATGAGCACCGTGATGGAAAT -3′; right primer 5′- AGCGTTACCGGTGTAGTCGT -3′) and *rrsA* (left primer, 5′- CGGTGGAGCATGTGGTTTAA -3′; right primer, 5′- GAAAACTTCCGTGGATGTCAAGA -3′) primers were run in quadruplicate for each strain. K-12 WT cDNA dilutions (2×, 4×, 10×, and 100×) were used to calculate the primer amplification efficiency.

### *tusBCD* knockout construction

The *ΔtusBCD* strain was constructed according to methods described elsewhere [60]. Primers were designed to knockout all 3 genes as one continuous fragment and replace it with the chloramphenicol acetyl transferase gene from pKD3. The upstream primer was 5′-TACATCCGCCAGTTCAAGAGCGGTGATTTCCAGGGGCAAGATAAGTAATGATGGGAATTAGCCATGGTCC-3′ and the downstream primer used was 5′-GTGTCAAGAAATATACAACGATCCCGCCATCACCAGGCCATCTGGCTGGGGTGTAGGCTGGAGCTGCTTCG-3′.

## Supporting Information

Figure S1 The raw *E. coli* growth time courses for the wild type strain and all of the knockout screens with lower-than-wild type infectivity. The gene missing from the strain is shown in the upper left corner of each graph. To facilitate comparison between strains cultured on the same plate, the plate number is indicated by the subscript (e.g., the *ΔyfiM* strain was cultured in Plate 6, and can be compared to the K-12 WT strain that was also cultured on this plate).
(1.25 MB PDF)

Figure S2 *E. coli* growth time courses for the K-12 WT strain and the knockout strains in Clusters 2–3 (except *cyaA* and *crr*) at three MOIs. These time courses have been non-dimensionalized with respect to growth rate and maximum growth capacity (as described in the main text) to facilitate comparison between the strains.
(0.42 MB PDF)

Figure S3 One hundred and twenty five simulated infection time courses using the computational model described in the main text. The parameter values for each time course can be determined from the legends (*k_i_* and *b*) and trace color (*f*). Additional parameter values were held constant at *k_s_* = 0, μ* = K* = 0.7. These simulations have been non-dimensionalized with respect to growth rate and maximum growth capacity (as described in the main text) to facilitate comparison between the overall simulation behaviors.
(0.63 MB PDF)

Figure S4 The complete set of clustered simulation time course derivatives. Parameter values for simulations are those used in Figure S3 (for additional details see Methods and main text).
(0.51 MB PDF)

Figure S5 *ΔtusBCD* infection dynamics. (A) Shows the infection dynamics of the [Fe-S] independent pathway strains along with three colonies from the *ΔtusBCD* construction. (B) 1 - Euclidian distance between time courses for knockouts in (A) are displayed.
(0.20 MB PDF)

Figure S6 Comparison of *E. coli* K-12 MG1655 with “Keio Collection” background strain BW25113. Solid lines indicate lambda phage infected samples. Dash lines indicate uninfected samples.
(0.11 MB PDF)

Table S1 Plaque assay results. Irregular lawn (il), small plaques (s), and zero plaques (0) are indicated for each strain.
(0.41 MB PDF)

Table S2 Human orthologues of lambda phage host-dependency genes.
(0.31 MB PDF)
