# Supplementary figures and images for: A Forward-Genetic Screen and Dynamic Analysis of Lambda Phage Host-Dependencies Reveals an Extensive Interaction Network and a New Anti-Viral Strategy
Source: PLoS Genet. 2010 Jul 8;6(7):e1001017. doi: 10.1371/journal.pgen.1001017 (PMC2900299; doi:10.1371/journal.pgen.1001017)

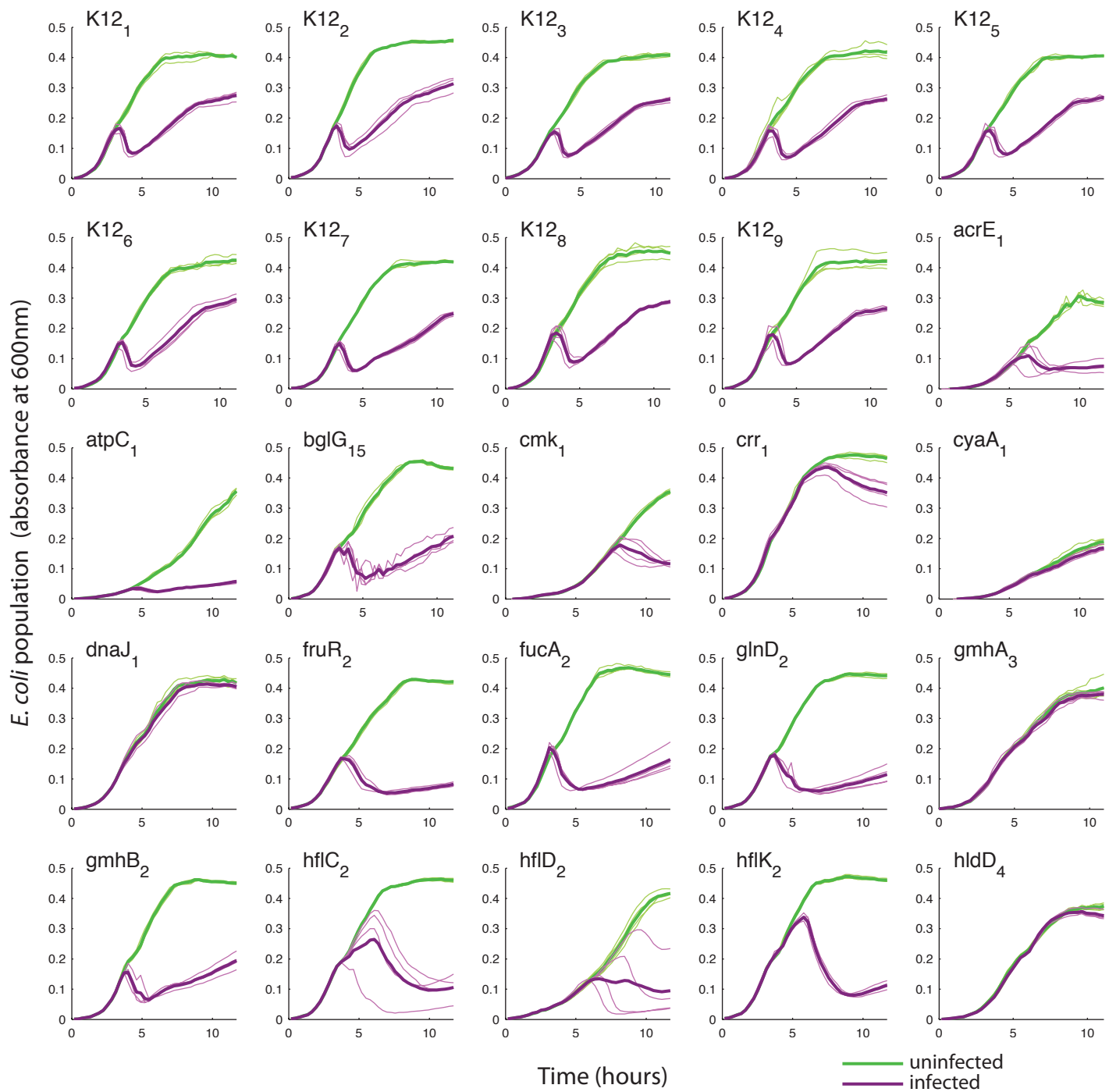

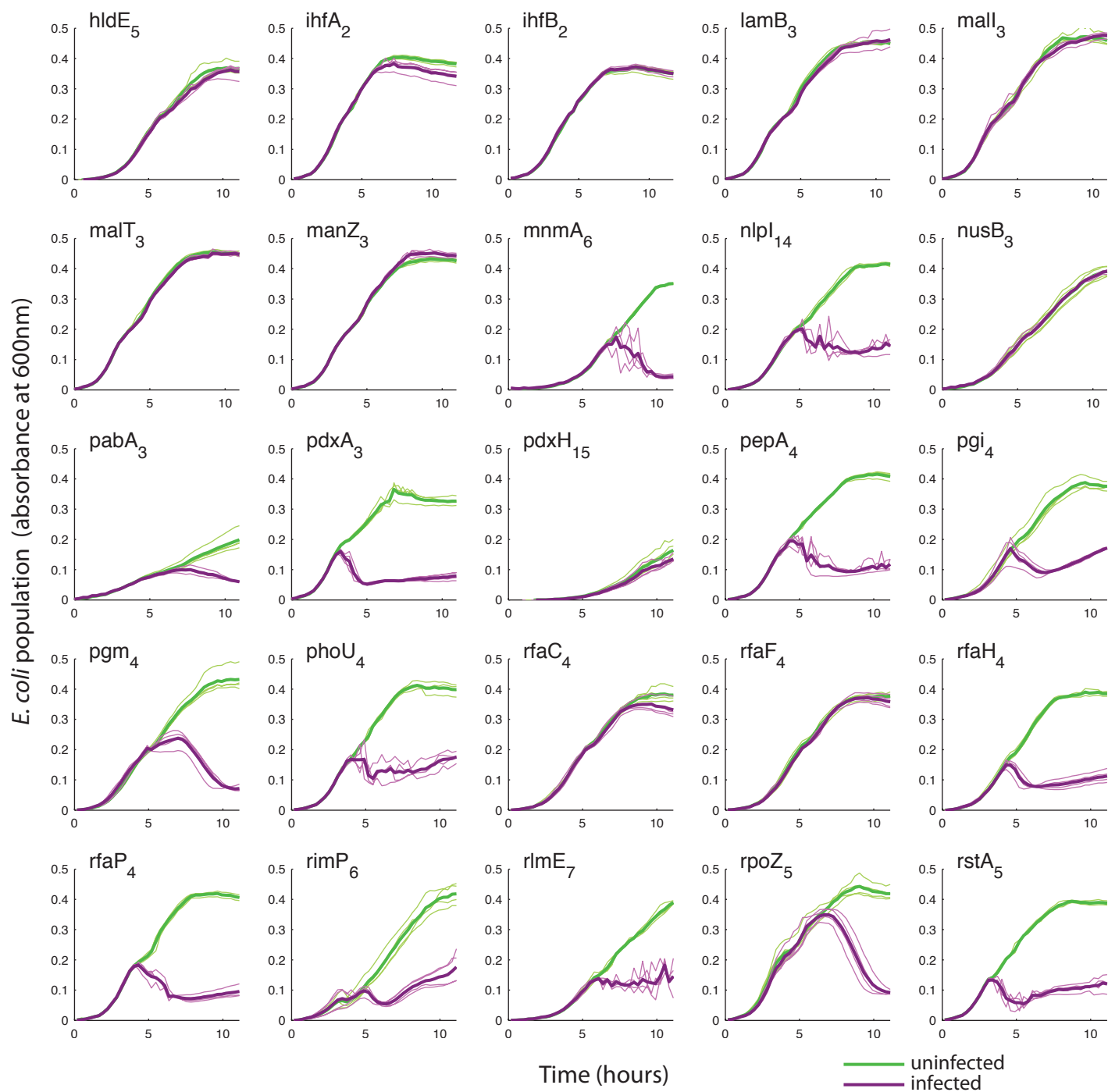

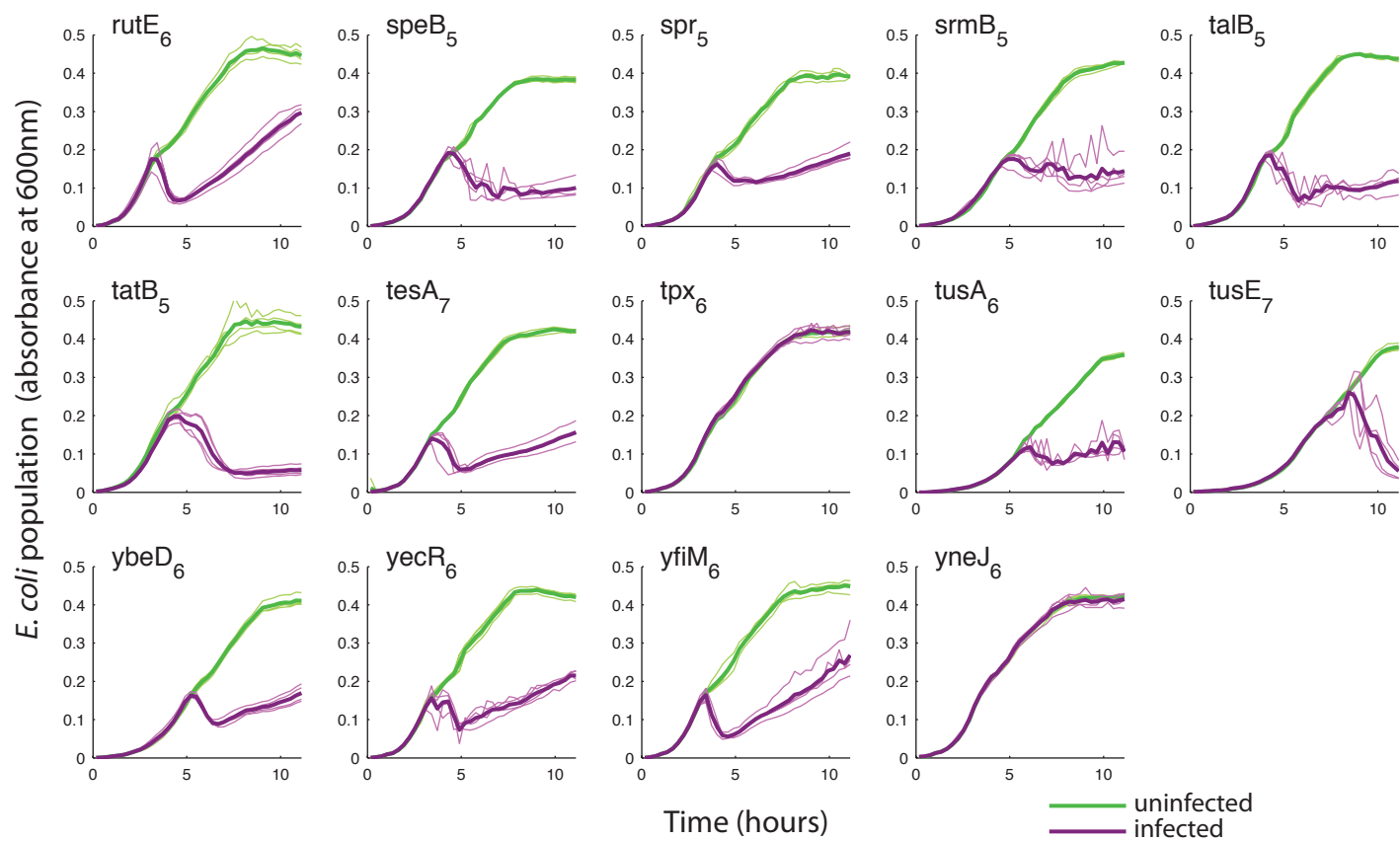

Supplement: Figure S1 — The raw E. coli growth time courses for the wild type strain and all of the knockout screens with lower-than-wild type infectivity. The gene missing from the strain is shown in the upper left corner of each graph. To facilitate comparison between strains cultured on the same plate, the plate number is indicated by the subscript (e.g., the ΔyfiM strain was cultured in Plate 6, and can be compared to the K-12 WT strain that was also cultured on this plate). (1.25 MB PDF) [file pgen.1001017.s001.pdf]

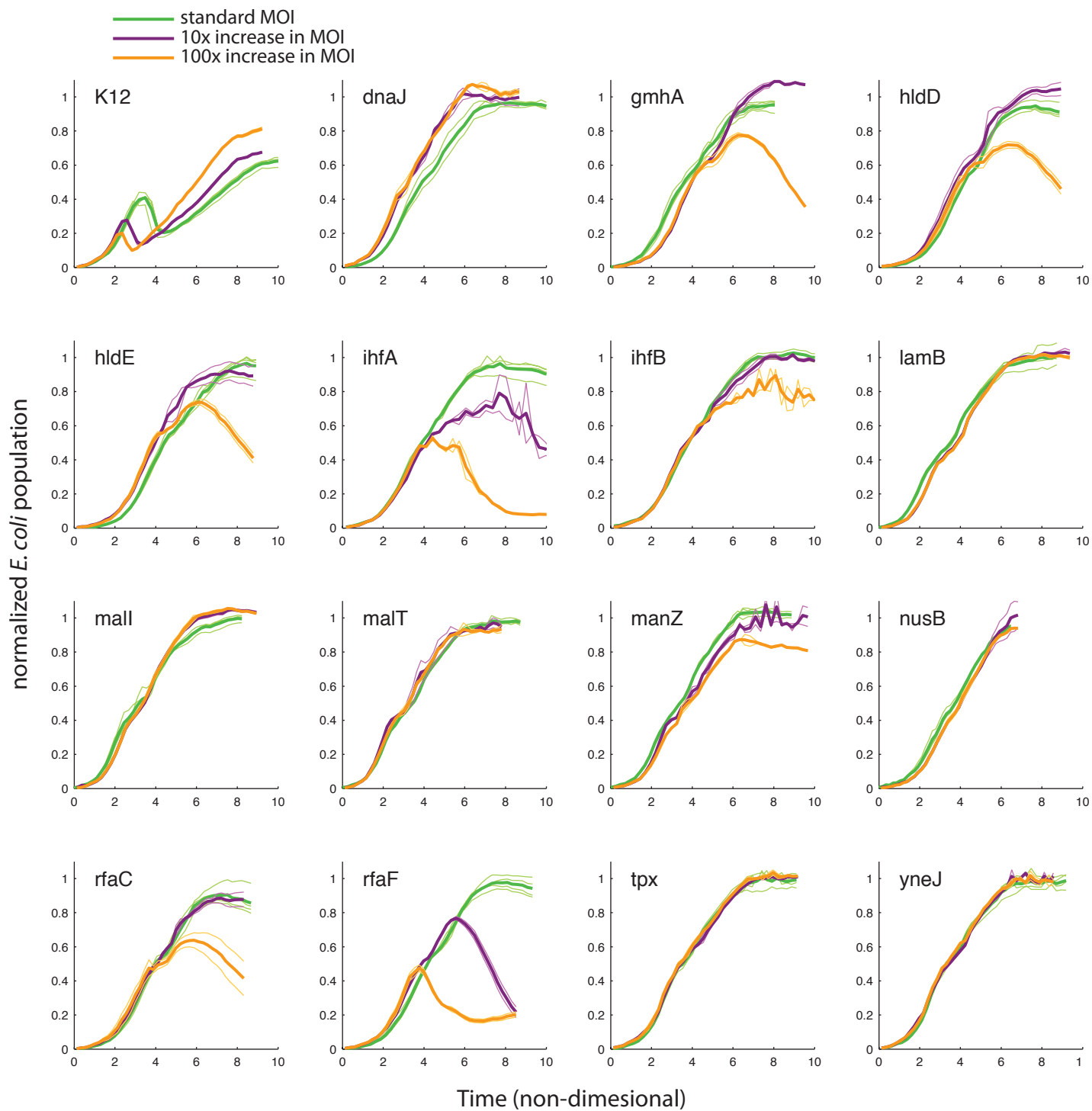

Supplement: Figure S2 — E. coli growth time courses for the K-12 WT strain and the knockout strains in Clusters 2–3 (except cyaA and crr) at three MOIs. These time courses have been non-dimensionalized with respect to growth rate and maximum growth capacity (as described in the main text) to facilitate comparison between the strains. (0.42 MB PDF) [file pgen.1001017.s002.pdf]

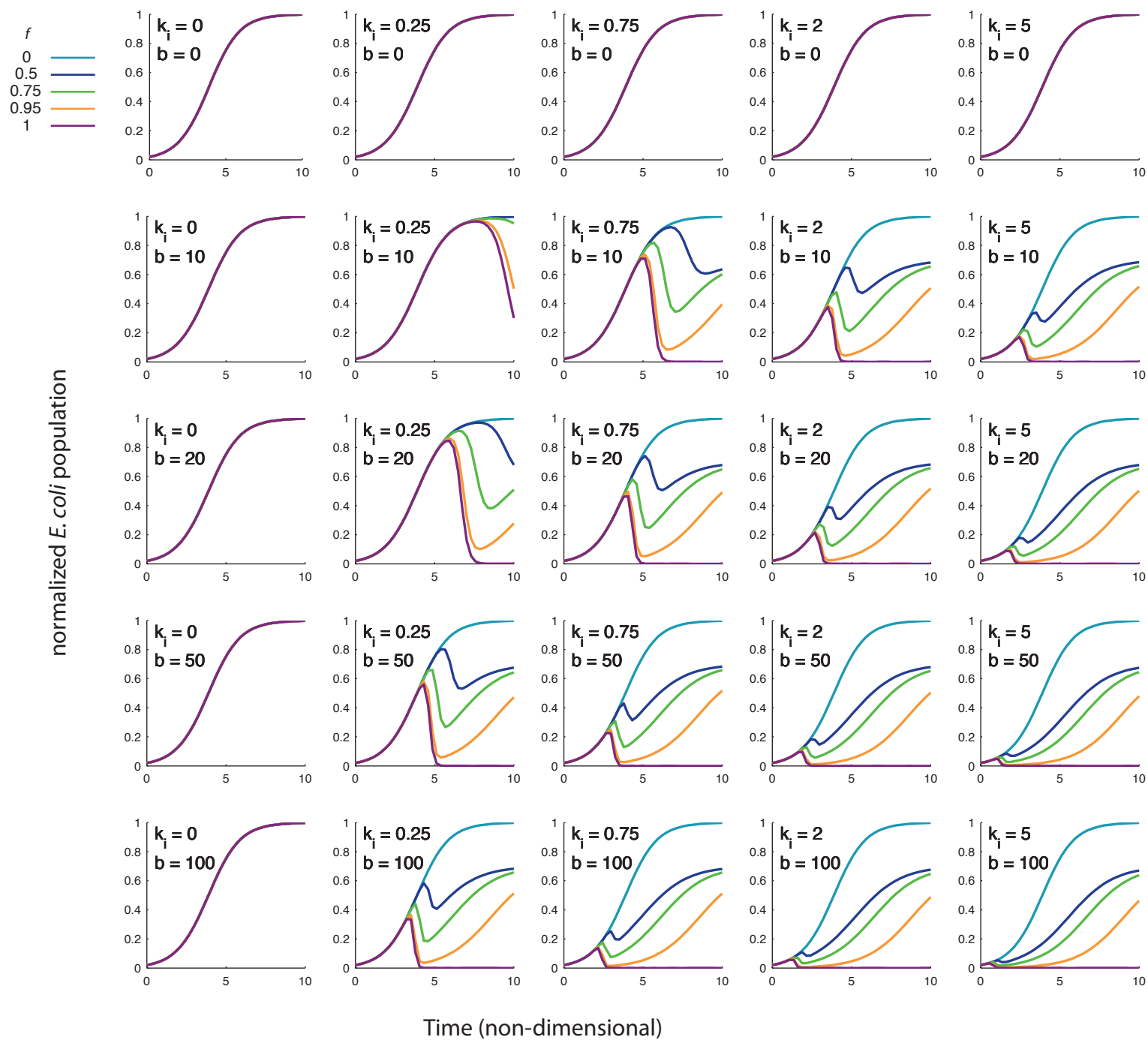

Supplement: Figure S3 — One hundred and twenty five simulated infection time courses using the computational model described in the main text. The parameter values for each time course can be determined from the legends (ki and b) and trace color (f). Additional parameter values were held constant at ks = 0, μ* = K* = 0.7. These simulations have been non-dimensionalized with respect to growth rate and maximum growth capacity (as described in the main text) to facilitate comparison between the overall simulation behaviors. (0.63 MB PDF) [file pgen.1001017.s003.pdf]

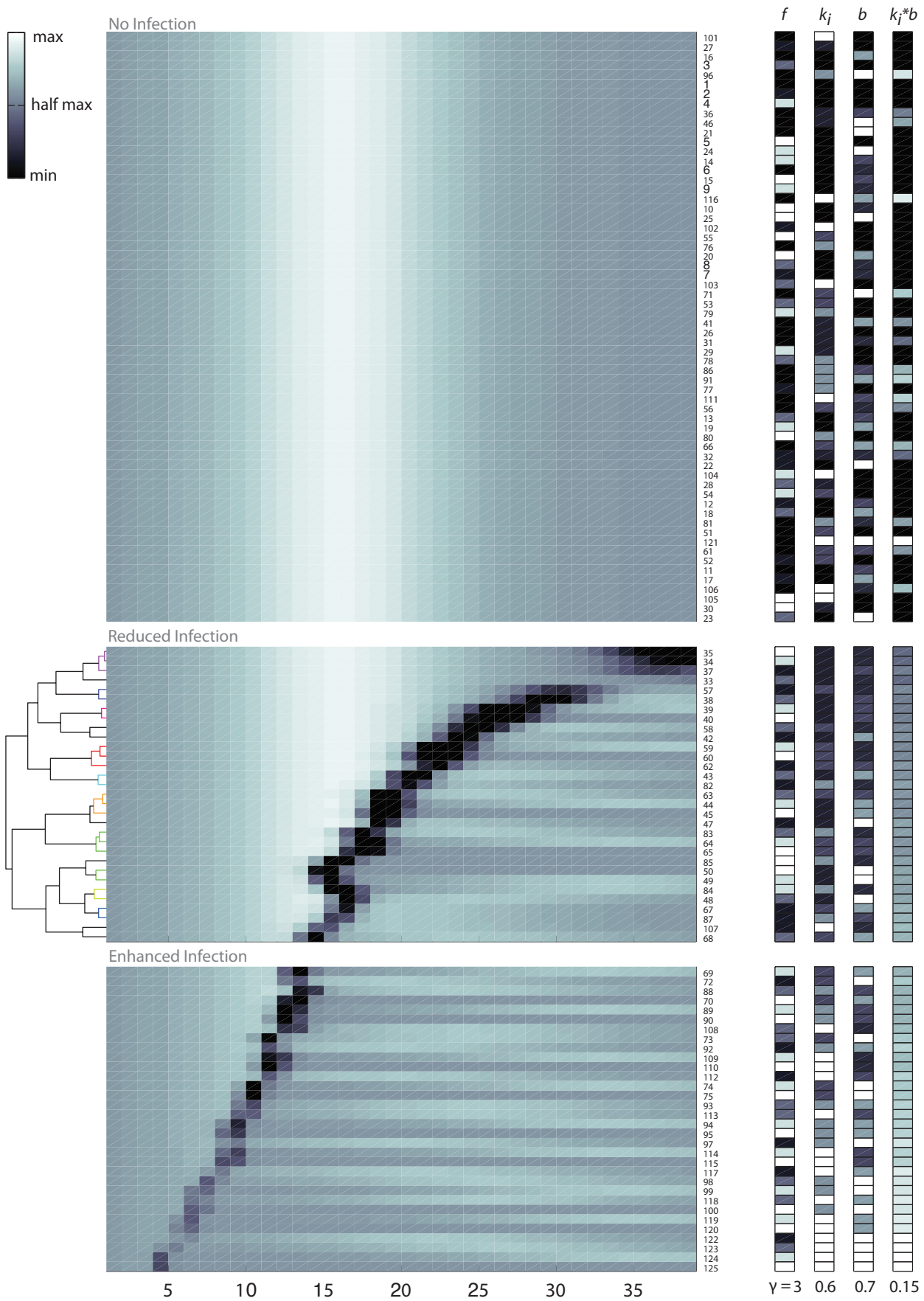

Supplement: Figure S4 — The complete set of clustered simulation time course derivatives. Parameter values for simulations are those used in Figure S3 (for additional details see Methods and main text). (0.51 MB PDF) [file pgen.1001017.s004.pdf]

A)

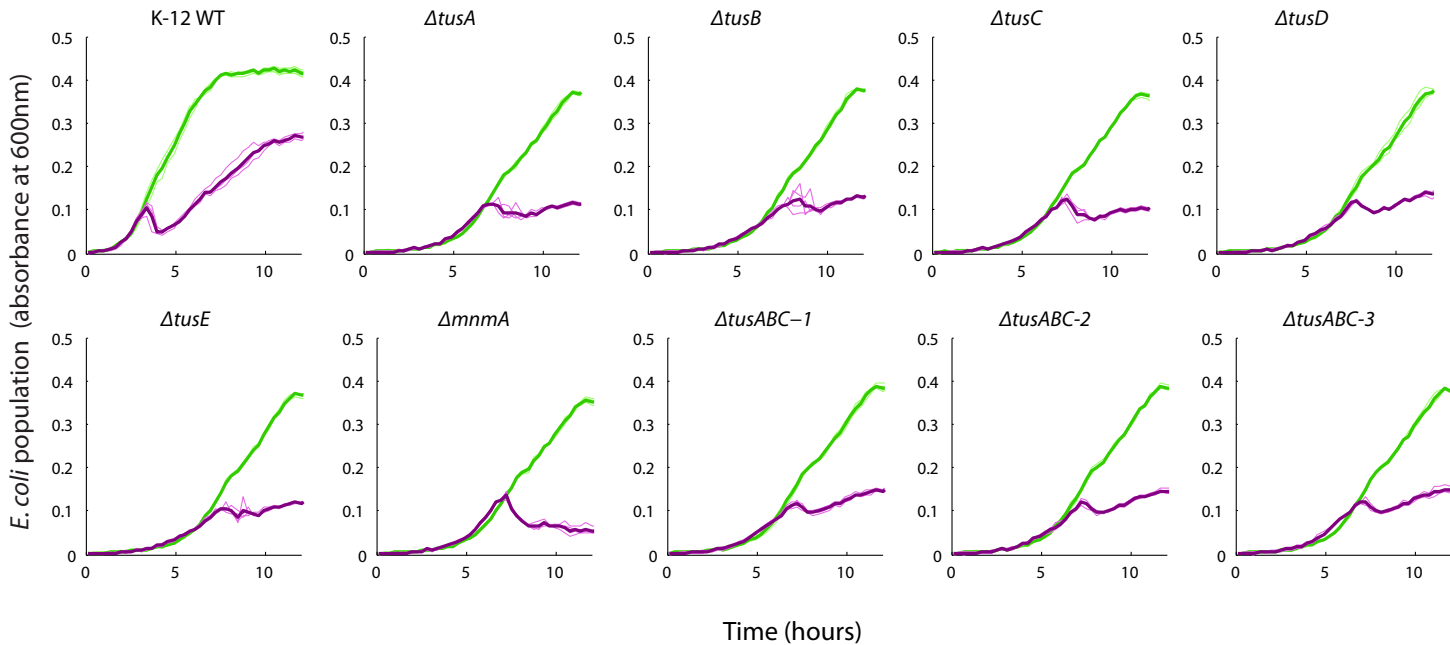

B)

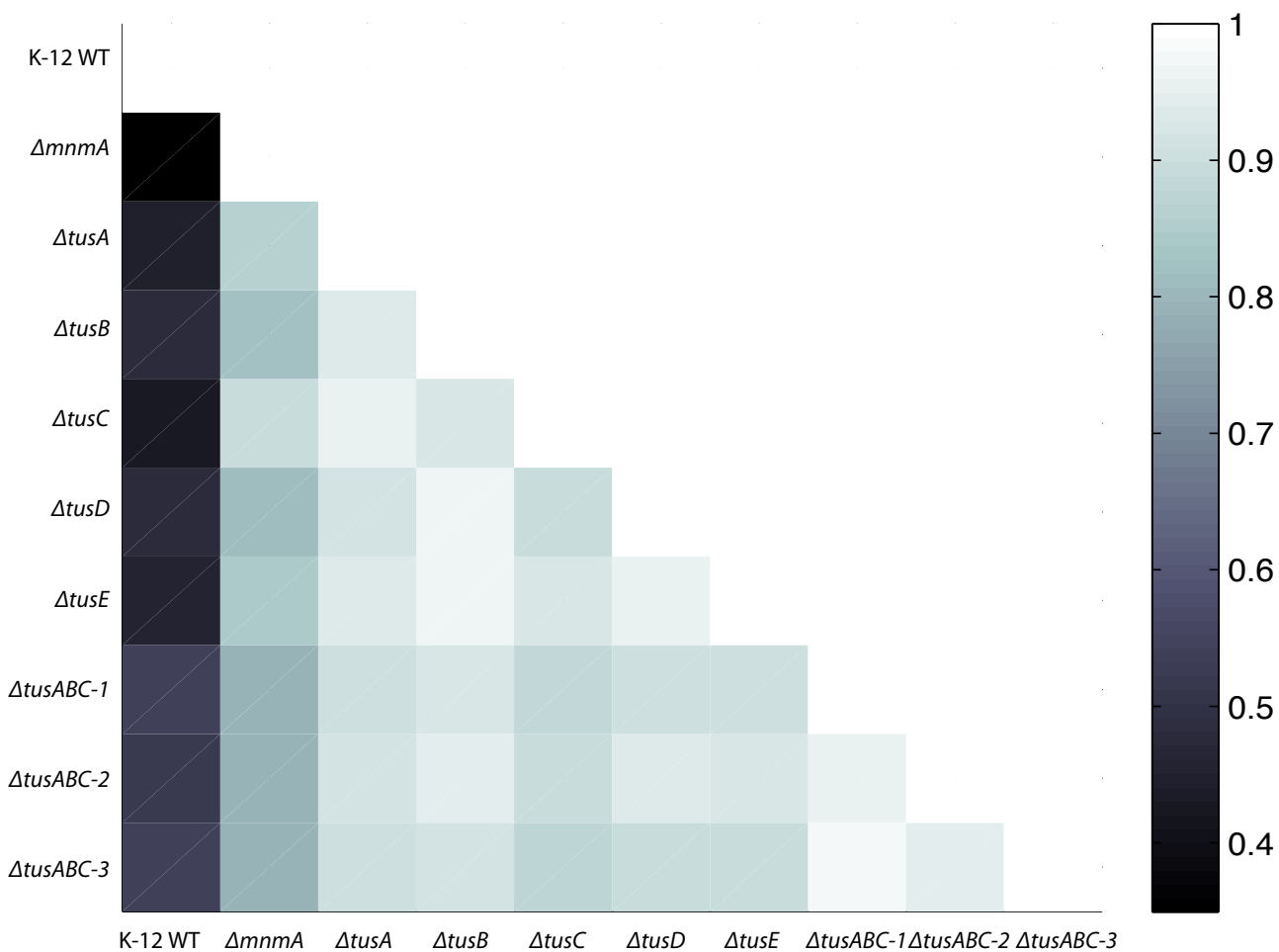

Supplement: Figure S5 — ΔtusBCD infection dynamics. (A) Shows the infection dynamics of the [Fe-S] independent pathway strains along with three colonies from the ΔtusBCD construction. (B) 1 - Euclidian distance between time courses for knockouts in (A) are displayed. (0.20 MB PDF) [file pgen.1001017.s005.pdf]

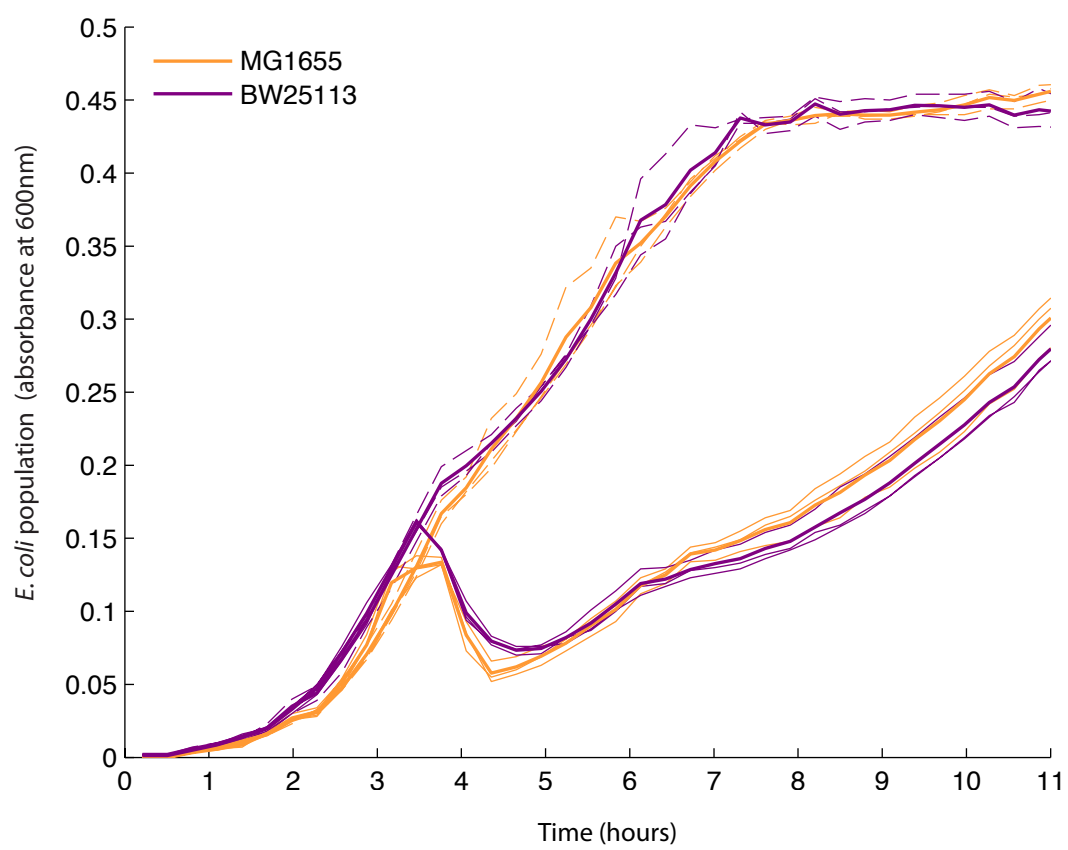

Supplement: Figure S6 — Comparison of E. coli K-12 MG1655 with “Keio Collection” background strain BW25113. Solid lines indicate lambda phage infected samples. Dash lines indicate uninfected samples. (0.11 MB PDF) [file pgen.1001017.s006.pdf]
