# Supplementary material for: A Forward-Genetic Screen and Dynamic Analysis of Lambda Phage Host-Dependencies Reveals an Extensive Interaction Network and a New Anti-Viral Strategy
Source: PLoS Genet. 2010 Jul 8;6(7):e1001017. doi: 10.1371/journal.pgen.1001017 (PMC2900299; doi:10.1371/journal.pgen.1001017)
Supplement: Table S1 — Plaque assay results. Irregular lawn (il), small plaques (s), and zero plaques (0) are indicated for each strain. (0.41 MB PDF) [file pgen.1001017.s007.pdf]

Gene name	Blattner #	Initial plaque	Validation plaque	Gene description
acrE	b3613	il	s	protease with a role in cell division
atpA	b3734	il	s	F1 sector of membrane-bound ATP synthase, alpha subunit
bglG	b3723	s	s	transcriptional antiterminator of the bgl operon
cmk	b0910	s	s	cytidylate kinase
crr	b2417	s	s	glucose-specific enzyme IIA component of PTS
cyaA	b3806	il	0	adenylate cyclase
dnaJ	b0015	0	0	chaperone Hsp40, co-chaperone with DnaK, DNA-binding
fruR	b0080	s	s	DNA-binding transcriptional dual regulator
fucA	b2800	s	s	L-fuculose-1-phosphate aldolase
glnD	b0167	il	s	uridylyltransferase
gmhA	b0222	0	s	D-sedoheptulose 7-phosphate isomerase
gmhB	b0200	0	s	D,D-heptose 1,7-bisphosphate phosphatase
hflC	b4175	s	s	modulator for HflB protease specific for phage lambda cII repressor
hflD	b1132	0	s	predicted lysogenization regulator
hflK	b4174	s	s	modulator for HflB protease specific for phage lambda cII repressor
hldD	b3619	0	s	ADP-L-glycero-D-mannoheptose-6-epimerase, NAD(P)-binding domain
hldE	b3052	il	s	fused heptose 7-phosphate kinase and heptose 1-phosphate adenyltransferase
ihfA	b1712	s	s	integration host factor (IHF), DNA-binding protein, alpha subunit
ihfB	b0912	0	s	integration host factor (IHF), DNA-binding protein, beta subunit
iscS	b2530	il	s	cysteine desulfurase (tRNA sulfurtransferase), PLP-dependent
lamB	b4036	0	0	maltose outer membrane porin (maltoporin)
malI	b1620	0	0	DNA-binding transcriptional repressor
malT	b3418	0	0	DNA-binding transcriptional activator, maltotriose-ATP-binding
manZ	b1819	0	0	mannose-specific enzyme IID component of PTS
mnmA	b1133	0	s	tRNA (5-methylaminomethyl-2-thiouridylate)-methyltransferase
nlpI	b3163	s	s	conserved protein
nusB	b0416	0	0	transcription antitermination protein
pabA	b3360	il	s	aminodeoxychorismate synthase, subunit II
pdxA	b0052	s	s	4-hydroxy-L-threonine phosphate dehydrogenase, NAD-dependent
pdxH	b1638	il	0	pyridoxine 5'-phosphate oxidase
pepA	b4260	s	s	aminopeptidase A, a cyteinylglycinase
pgi	b4025	0	s	glucosephosphate isomerase
pgm	b0688	0	s	phosphoglucomutase
phoU	b3724	s	s	DNA-binding transcriptional regulator
rfaC	b3621	0	0	ADP-heptose:LPS heptosyl transferase I
rfaF	b3620	s	0	ADP-heptose:LPS heptosyltransferase II
rfaH	b3842	s	s	DNA-binding transcriptional antiterminator
rfaP	b3630	0	s	kinase that phosphorylates core heptose of lipopolysaccharide
rimP	b3170	0	s	conserved protein
rlmE	b3179	s	s	23S rRNA methyltransferase
rpoZ	b3649	0	s	RNA polymerase, omega subunit
rstA	b1608	s	s	DNA-bindng response regulator in two-component regulatory system with RstB
rutE	b1008	s	s	predicted nitroreductase
speB	b2937	0	s	agmatinase
spr	b2175	0	0	predicted peptidase, outer membrane lipoprotein
srmB	b2576	0	s	ATP-dependent RNA helicase
talB	b0008	s	s	transaldolase B
tatB	b3838	s	s	TatABCE protein translocation system subunit
tesA	b0494	s	s	multifunctional acyl-CoA thioesterase I and protease I and lysophospholipase L1
thyA	b2827	il	0	thymidylate synthetase
tpx	b1324	0	s	lipid hydroperoxide peroxidase
tusA	b3470	0	s	mediator of IscS desulfur transfer, 2-thiolation step of mnm(5)-s(2)U34-tRNA synthesis
tusE	b0969	0	s	tRNA 2-thiouridine synthesizing protein, sulfur mediator, TusE
ybeD	b0631	0	s	conserved protein
yecR	b1904	0	s	predicted protein
yfiM	b2586	s	s	predicted protein
yneJ	b1526	0	0	predicted DNA-binding transcriptional regulator
