# Supplementary material for: A Forward-Genetic Screen and Dynamic Analysis of Lambda Phage Host-Dependencies Reveals an Extensive Interaction Network and a New Anti-Viral Strategy
Source: PLoS Genet. 2010 Jul 8;6(7):e1001017. doi: 10.1371/journal.pgen.1001017 (PMC2900299; doi:10.1371/journal.pgen.1001017)
Supplement: Table S2 — Human orthologues of lambda phage host-dependency genes. (0.31 MB PDF) [file pgen.1001017.s008.pdf]

Gene Name	Blattner #	KEGG Ortholog #	Human Ortholog
acrE	b3613	0	
atpA	b3734	K02111	
bglG	b3723	K03488	
cmk	b0910	K00945	CMPK1
crr	b2417	K02777	
cyaA	b3806	K05851	
dnaJ	b0015	K03686	
fruR	b0080	K03435	
fucA	b2800	K01628	
glnD	b0167	K00990	
gmhA	b0222	K03271	
gmhB	b0200	K03273	
hflC	b4175	K04087	
hflD	b1132	K07153	
hflK	b4174	K04088	
hldD	b3619	K03274	
hldE	b3052	K03272	
ihfA	b1712	K04764	
ihfB	b0912	K05788	
iscS	b2530	K04487	NFS1
lamB	b4036	K02024	
lexA	b2175	0	
malI	b1620	0	
malT	b3418	K03556	
manZ	b1819	K02796	
mnmA	b1133	K00566	TRMU
nlpI	b3163	K05803	
nusB	b0416	K03625	
pabA	b3360	K01664	
pdxA	b0052	K00097	
pdxH	b1638	K00275	PNPO
pepA	b4260	K01255	
pgi	b4025	K01810	GPI
pgm	b0688	K01835	PGM1; PGM2
phoU	b3724	K02039	
rfaC	b3621	K02841	
rfaF	b3620	K02843	
rfaH	b3842	K05785	
rfaP	b3630	K02848	
rimP	b3170	K09748	
rlmE	b3179	K02427	FTSJ1
rpoZ	b3649	K03060	
rstA	b1608	K07661	
rutE	b1008	K09019	
speB	b2937	K01480	AGMAT
srmB	b2576	K05590	
talB	b0008	K00616	TALDO1
tatB	b3838	K03117	
tesA	b0494	K10804	
thyA	b2827	K00560	TYMS
tpx	b1324	K11065	
tusA	b3470	K04085	
tusE	b0969	K11179	
ybeD	b0631	K09158	
yecR	b1904	0	
yfiM	b2586	K05811	
yneJ	b1526	0	
